# Embryonic expression of a Long Toll (Loto) gene in the onychophorans *Euperipatoides kanangrensis* and *Cephalofovea clandestina*

**DOI:** 10.1007/s00427-018-0609-8

**Published:** 2018-05-26

**Authors:** Ralf Janssen, Linushiya Lionel

**Affiliations:** Department of Earth Sciences, Palaeobiology, Uppsala University, Villavägen 16, 75236 Uppsala, Sweden

**Keywords:** Convergent extension, Toll, Loto, Segmentation, Onychophora

## Abstract

Recent research has shown that Toll genes, and in particular a newly defined class of Toll genes, the so-called Long Toll Genes (Loto genes), are crucial factors in embryogenesis. In arthropods, they are involved in axis formation via a process called convergent extension (CE). A hallmark of Loto genes is their relatively (compared to other Toll genes) high number of leucine-rich repeat elements (LRRs) coupled with the fact that they are expressed in transverse stripes in all segments, or a subset of segments, patterns that are reminiscent of classical segmentation genes such as the pair-rule genes. Onychophorans represent a close outgroup to the arthropods; however, their embryonic development differs substantially. It is unclear if convergent extension contributes to onychophoran germ band formation and, if so, whether Loto genes are involved in governing this process. This study identifies a single onychophoran Toll gene from a sequenced embryonic transcriptome in two onychophoran species. The identified gene shows sequence and expression pattern characteristics of Loto genes. However, its expression pattern also comprises some general differences to arthropod Loto genes that are involved in CE.

**Electronic supplementary material:**

The online version of this article (10.1007/s00427-018-0609-8) contains supplementary material, which is available to authorized users.

## Introduction

Convergent extension (CE) is a conserved developmental process during animal development that is often involved in elongation of the anterior-to-posterior body axis (AP axis). CE is characterized by directional and synchronized intercalation of cells which subsequently leads to the elongation of the embryo (e.g., Keller et al. 2000; Zallen and Wieschaus 2004; Rauzi et al. 2008; Shindo 2017). In the main arthropod model organism, the vinegar fly *Drosophila melanogaster*, this process is under control of the primary pair rule genes *even-skipped* (*eve*) and *runt* (*run*), and as such, CE is linked to AP axis segmentation (e.g., St Johnston and Nüsslein-Volhard 1992; Irvine and Wieschaus 1994; Zallen and Wieschaus 2004). Recently, it has been shown that some Toll receptor-encoding genes are involved in CE under control of Eve and Run (Paré et al. 2014). Both *Toll-8* (aka *Tollo*) and *Toll-2* (aka *18-wheeler*) were upregulated in the absence of the transcriptional repressors Eve and Run (Paré et al. 2014). The *Drosophila* Toll genes, *Toll-2*, *Toll-6*, *Toll-7*, and *Toll-8* are all expressed in transverse stripes reminiscent of the expression patterns of the pair-rule genes *eve* and *run* (Chiang and Beachy 1994; Kambris et al. 2002), and it has been shown that Toll receptors are generally involved in cell adhesion and cell migration (e.g., Keith and Gay 1990; Wang et al. 2005; Kleve et al. 2006), mechanisms that are crucial for CE. Toll genes encode single-pass transmembrane glycoproteins containing a number of extracellular leucine-rich repeats (LRRs) and a conserved cytoplasmic Toll/interleukin-1 receptor (TIR) domain (reviewed in Chang 2010). These genes are mostly known for their conserved function in regulating innate immunity in various animal groups including arthropods (reviewed in, e.g., Kawai and Akira 2010; Imler 2014).

A recent comprehensive study covering all main branches of arthropods, i.e., Hexapoda, “Crustacea,” Myriapoda, and Chelicerata, has shown that the general function of Toll genes in CE is conserved in Arthropoda as a whole (Benton et al. 2016). In their study, at least one Toll gene has been identified that has a likely (based on conserved gene expression) or proven (by means of RNAi interference experiments) function in CE in each investigated species (Benton et al. 2016). The Benton et al. (2016) study shows that Toll genes that are likely involved in CE are “longer” and possess a higher number of LRRs than Toll genes that fall outside this group. Therefore, and because of the likely conserved role in CE, these Toll genes were named “Long Toll” genes (abbreviated as “Loto” genes) (Benton et al. 2016).

The aim of this study is to investigate the presence and possible function of potential Loto class genes in onychophorans. Onychophora comprises the likely sister group (or at least a very close outgroup) to Arthropoda (Campbell et al. 2011). This current study is thus the continuation of the work started by Paré et al. (2014) and Benton et al. (2016) to investigate the evolutionary origin of the role that Toll/Loto genes play in CE. It also addresses the question of whether CE is involved in germ band formation in onychophorans. Although this is the case for many animal groups, and likely is the case for all (investigated) arthropods, Onychophora comprises another mode of embryogenesis and it is not clear if CE is involved in this process.

We also investigated Loto class genes in a myriapod, the millipede *Glomeris marginata*, representing the second main branch of Myriapoda (the study by Benton et al. (2016) investigated Loto gene expression in the centipede *Strigamia maritima*).

## Methods

### Animal husbandry and embryo preparation

Mature specimens of *Glomeris marginata* were collected in the Reichswald Forest (Germany/NRW), and were kept in plastic containers filled with decomposing beech leaves as food and moist clay as building material for egg-chambers. Animals were kept at a constant temperature of 21–22 °C. Eggs were removed from the egg chambers by hand. The chorion was removed with bleach (prior to fixation), and the vitelline membrane was removed by hand with fine forceps (after fixation). Embryos were fixed for approximately 4–6 h in 4% formaldehyde in PBST and heptane. Fixed embryos were stored in methanol at − 20 °C.

Pregnant females of *Euperipatoides kanangrensis* and *Cephalofovea clandestina* were collected in the Kanangra-Boyd National park in the Blue Mountains, north of Sydney/Australia. Embryos were removed by dissecting the females, and the chorion and vitelline membrane were removed with fine forceps. Embryos were fixed and stored in the same way as described for *Glomeris* (see above).

### Gene cloning and probe synthesis

RNA isolation and cDNA synthesis were performed as per Janssen et al. (2004). Gene fragments were amplified via RT-PCR with gene-specific primers based on sequenced embryonic transcriptomes (Janssen and Budd 2013; Janssen and Posnien 2014) (see Supplementary Table 1 for primer sequences). The *Cephalofovea* gene fragment was isolated using gene-specific primers based on the sequence of *Euperipatoides LotoA*. For the amplification of this fragment, first an initial PCR was performed, followed by a second PCR using a second internal (nested) set of primers. All fragments were cloned into the pCRII-TOPO vector (Invitrogen).

Sequences of all gene fragments were determined by sequencing (Big Dye Terminator Cycle Sequencing Kit; Perkin-Elmer Applied Biosystems, Foster City, CA, USA) on an automatic analyzer (ABI3730XL; Perkin-Elmer Applied Biosystems) by a commercial sequencing service (Macrogen, Seoul, Korea). Sequences are available in GenBank under the accession numbers listed in Supplementary Table 2. DIG-labeled RNA probes were synthesized with either Sp6 or T7 RNA polymerase (ROCHE). Probes were purified with the RNeasy Mini kit (QIAGEN) prior to whole-mount in situ hybridization.

### Whole-mount in situ hybridization and nuclear staining

We used an improved whole-mount in situ hybridization (WISH) protocol for *Euperipatoides* and *Cephalofovea* embryos that is described in Supplementary Text 1. Major changes to previous protocols, as used for *Glomeris* (see supplementary material in Janssen et al. 2015), include a treatment of embryos with H_2_O_2_ prior to rehydration, and the use of 5% dextran sulfate in the hybridization buffer (see Lauter et al. 2011). All embryos were stained with BM Purple for 2 to 4 h, except for embryos used in confocal microscopy; these embryos were stained for 48 h in FastRed (staining solution was changed every 8 h). Cell nuclei were visualized by incubation in 1 μg/ml of the fluorescent dye 4-6-diamidino-2-phenylindole (DAPI) in phosphate-buffered saline with 0.1% Tween-20 (PBST) for 20 min.

### Sequence analysis

The complete (as far as available) open reading frames of Toll/Loto genes were aligned using ClustalX with default parameters in MacVector v12.6.0 (MacVector, Inc., Cary, NC).

A Bayesian phylogenetic analysis was performed with MrBayes (Huelsenbeck and Ronquist 2001) using a fixed WAG amino acid substitution model with gamma-distributed rate variation across sites (with four rate categories). An unconstrained exponential prior probability distribution on branch lengths and an exponential prior for the gamma shape parameter for among-site rate variation was applied. The final topology was estimated using 600,000 cycles for the MCMCMC (metropolis-coupled Markov chain Monte Carlo) analysis with four chains and the chain-heating temperature set to 0.2. The Markov chain was sampled every 200 cycles. Clade support was assessed with posterior probabilities computed with MrBayes. An internet-based platform (http://lrrsearch.com/index.php?page=tool) was used to search for leucine-rich repeats (LRR) (Bej et al. 2014) (Supplementary Table 3). Toll/interleukin-1 receptor domains (TIR) were identified using Blast search.

### Data documentation

Bright field and DAPI pictures were taken with a Leica DC100 digital camera under a Leica dissection microscope. For confocal microscopy, we used an inverted Leica SP5 confocal microscope. Optical sections were taken every 6.5 μm. Brightness, contrast, and color values were corrected using image-processing software (Adobe Photoshop CC for Apple Macintosh; Adobe Systems Inc. San Jose, CA, USA). For the better documentation of *Ek-LotoA* expression, stained embryos were embedded in 2% of low-melting agarose, and cut with sharpened tungsten needles.

## Results

### Phylogenetic analysis

**Fig. 1:**
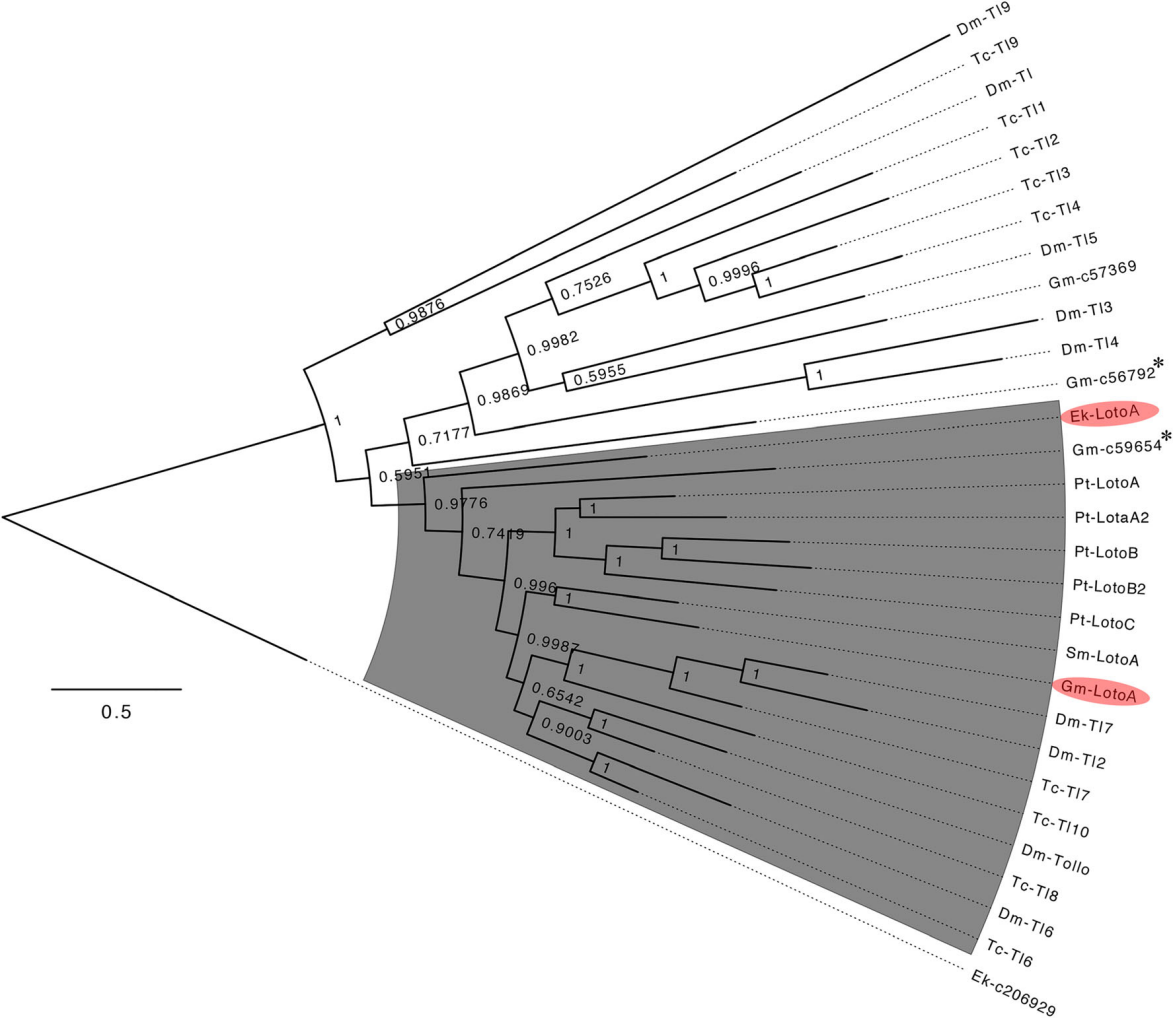
Bayesian inference analysis showing the distribution of panarthropod Toll and Loto genes. The gray shade indicates Loto class Toll genes. Loto genes investigated in this study are highlighted with red shades. Posterior probabilities > 0.5 are indicated. The onychophoran *slit* gene (*Ek-c206929*) serves as outgroup. Asterisks mark genes with incomplete sequence information. Note that Loto and Toll genes of *Drosophila* and *Tribolium* (based on the analysis in Benton et al. (2016)) have been used to calculate the tree, except for *Parasteatoda* and *Strigamia* for which only confirmed Loto genes were used to construct the tree. See text for further information. Species abbreviations: Dm, *Drosophila melanogaster* (Hexapoda: Diptera); Ek, *Euperipatoides kanangrensis* (Onychophora); Gm, *Glomeris marginata* (Myriapoda: Diplopoda); Pt, *Parasteatoda tepidariorum* (Chelicerata: Araneae); Sm, *Strigamia maritima* (Myriapoda: Chilopoda); Tc, *Tribolium castaneum* (Hexapoda: Coleoptera)

We identified a single *Euperipatoides* Toll gene from a sequenced embryonic transcriptome. Although overall coverage of this transcriptomic data set appears to be very good as indicated by the fact that none of the expected key developmental genes are missing (e.g., Janssen and Budd 2013; Hogvall et al. 2014; Janssen et al. 2014), it is however possible that our transcriptome data does not cover all genes. It is also possible that further Toll/Loto genes are expressed at earlier or later developmental stages. In our phylogenetic analysis, this onychophoran Toll gene clusters with the confirmed arthropod Loto class genes (Fig. 1). It also possesses a large number of LRRs (as well as a TIR domain typical for Toll genes), as is also the case for arthropod Loto genes (Benton et al. 2016) (Supplementary Table S3). We therefore name this gene *Ek-LotoA*. The obtained fragment of *Cephalovofea* (named *Cc-LotoA*) is more than 99% (404 of 407 amino acids) identical on the protein level with that of *Ek-LotoA*.

We identified several Toll genes in the sequenced embryonic transcriptome of *Glomeris*, one of which clusters with high support with *Strigamia LotoA* (Fig. 1). We therefore name this gene *Glomeris LotoA* (*Gm-LotoA*). We also identified three more Toll genes, of which two are represented by incomplete sequence information (i.e., *Gm-c59654*, *Gm-c56792*). One of these, *Gm-c59654*, clusters with arthropod Loto genes, the other, *Gm-c56792*, clusters with arthropod non-Loto Toll genes (Fig. 1). We did not name these genes according to our phylogenetic analysis, since we lack complete sequence information of these genes, and thus their true phylogenetic position remains unclear. One *Glomeris* gene, *Gm-c57369*, clusters confidentially with arthropod non-Loto Toll genes (Fig. 1).

### Expression patterns

#### *Euperipatoides* and *Cephalofovea LotoA*

**Fig. 2:**
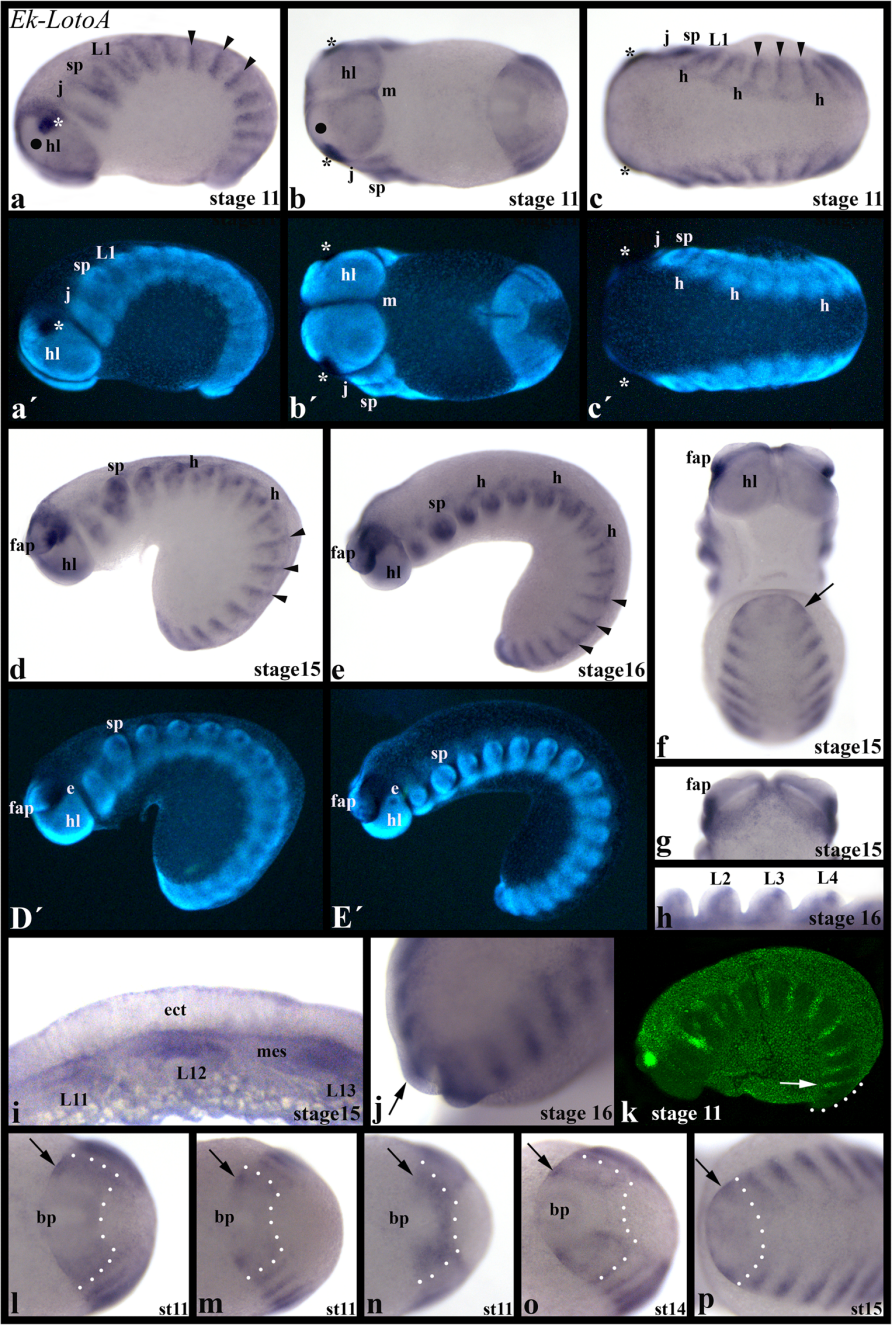
Expression of *Euperipatoides kanangrensis LotoA.* In (a–e, h, i, k) anterior is to the left. In (f, g) anterior is up. (j) On top view on the posterior end of the embryo; the embryo is slightly tilted. In (l–p), anterior is to the right. (a) Lateral view. Segmental stripes of expression are indicated by arrowheads. Expression in the dorsal of the head lobes and the frontal appendages is indicated by an asterisk. The filled circle marks the median region of the head lobes that does not express *LotoA*. (b) Ventral view. Filled circle and asterisks as in (a). (c) Dorsal view. Arrowheads and asterisks as in (a). (d) Lateral view. Arrowheads as in (a). (e) Lateral view. Arrowheads as in (a). (f) Ventral view. Arrow points to expression in (or near) the segment addition zone (SAZ). (g) Dorsal view on the head (same embryo as in (f)). (h) Ventral view. Close up on walking limbs. (i) Section through tissue expressing *Ek-LotoA* in periodic transverse stripes. Note that expression is in the mesoderm but not (or only very weakly) in the overlaying ectoderm. (j) Dorsal view. Arrow points to ectodermal expression in the SAZ. (k) Confocal Z-stack (see suppl. Figure S2 for further information and single optical sections). The arrow points to the most posterior mesodermal stripe of expression. Note that the weak expression in the saz (dotted line; cf. panels (l–p)) is not detectable by confocal microscopy and FastRed staining. (l–p) The arrows point to dynamic patterns of ectodermal expression in the SAZ (dotted lines). Developmental stages (after Janssen and Budd (2013)) are indicated. (a´–e´) represent DAPI counter stained embryos shown in (a–e). e, eye; ect, ectoderm; fap, frontal appendages; h, heart (dorsal tube); hl, head lobe; j, jaw; L1, first walking limb; m, mouth; mes, mesoderm; sp, slime papilla

For *Euperipatoides*, we investigated embryos of the developmental stages 9–16, and for *Cephalofovea*, we investigated stages 10–19 (after Janssen and Budd 2013) (Suppl. Table S4). We observed that the expression profile of both genes, *Ek-LotoA* and *Cc-LotoA*, is identical in the investigated developmental stages. *LotoA* is transiently expressed in transverse stripes in all formed segments (Fig. 2 and Suppl. Fig. S1). The segmental stripes of *LotoA* expression are mesodermal (Fig. 2 (i, j) and Suppl. Fig. S2). However, in tissue near the posterior segment addition zone (SAZ), *LotoA* appears to be expressed in a dynamic pattern in ectodermal tissue (Fig. 2 (f, j, l–p), and Suppl. Fig. S1B, C). We tried to further investigate this pattern by means of confocal microscopy using FastRed as a marker. However, since this expression is weak, and the sensitivity of FastRed relatively low (see Lauter et al. 2011), we could not detect this signal by means of fluorescent in situ hybridization; the stronger, mesodermal signal and the strong expression in the head lobes, however, are clearly recognizable in FastRed stained embryos (Fig. 2 (k) and Suppl. Fig. S2).

Persisting expression is in the dorsal region of the head lobes including the posterior part of the frontal appendages (Fig. 2(a–g, k) and Suppl. Fig. 1A–C), and in younger embryos in the ventral part of the head lobes (Fig. 2 (a, b)), tissue that likely contributes to the brain (cf. expression of *Notch*, *Delta* and *achaete-scute* (Eriksson and Stollewerk 2010; Janssen and Budd 2016)). This latter expression is in the form of a salt and pepper pattern. The median region of the head lobes does not express *LotoA* (Fig. 2 (a, b) and Suppl. Fig. S1A, C). *LotoA* is expressed at the dorsal edge of the segments, tissue that will likely contribute to the heart (dorsal tube, cf. expression of the heart marker *H15* (Janssen et al. 2015)) (Fig. 2 (c–e) and Suppl. Fig. S1A). The outgrowing limb buds express *LotoA*; this expression is first only mesodermal, but at later developmental stages, ectodermal cells near the tips of all appendages except for the jaws and the frontal appendages express *LotoA* as well (Fig. 2 (d, e, h) and Suppl. Fig. S1D).

In late developmental stages that were not investigated in *Euperipatoides*, we find strong expression of *Cc-LotoA* in the head and the limbs. Tissue between the limbs express *Cc-LotoA* only weakly (Supp. Fig. S2A).

#### *Glomeris LotoA*

**Fig. 3:**
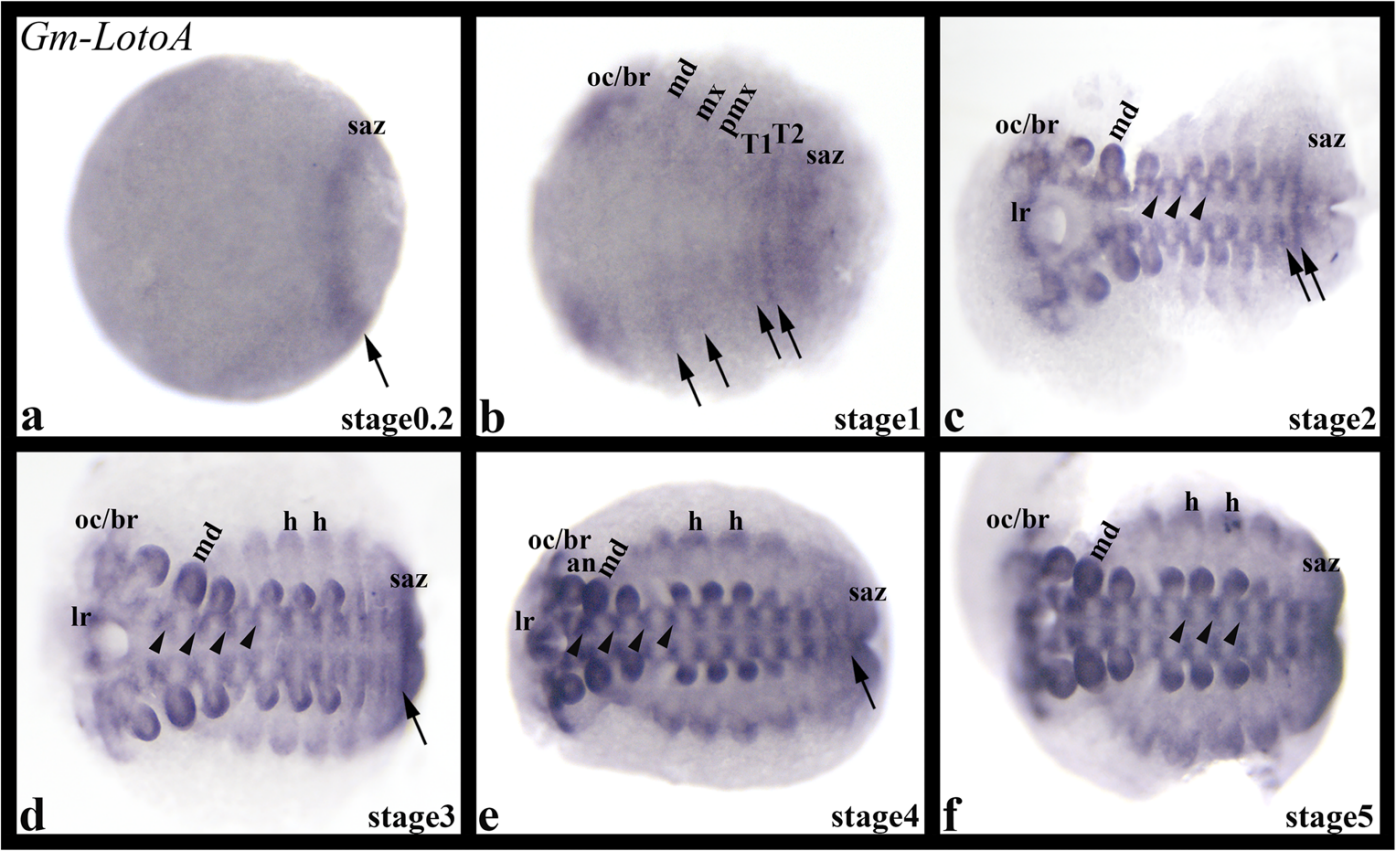
Expression of *Glomeris marginata LotoA.* In all panels, anterior is to the left representing ventral views. Arrows point to expression in transverse segmental stripes. Arrowheads point to expression in the ventral nervous system. an, antenna; h, heart; lr, labrum; mx, maxilla; md, mandible; oc/br, ocular region/brain; pmx, postmaxillary segment; saz, segment addition zone

*Gm-LotoA* is first expressed at stage 0.2 in the form of a single transverse domain in (or anterior adjacent to) the segment addition zone (Fig. 3a). At subsequent developmental stages, transient transverse stripes appear in an irregular pattern in the anterior segments formed from the regio germinalis (blastoderm) (cf. Janssen et al. 2004) (Fig. 3b). Transverse transient stripes form in nascent posterior segments (Fig. 3b–e). These stripes transform into patch-like expression in the ventral nervous system (Fig. 3c–f). Additional expression appears in the dorsal of the head lobes/brain (Fig. 3b–f), the limbs including the labrum (Fig. 3c–e), and the developing heart tube at the dorsal edges of the embryo (Fig. 3d–f).

#### Other *Glomeris* Toll genes, *Gm-c56762* and *Gm-c59654*

According to our analysis, both genes are either expressed ubiquitously at a low level, or are not expressed at the investigated embryonic stages (data not shown).

## Discussion

### Expression of a single Toll gene during onychophoran embryogenesis

We discovered only one Toll gene, a Loto class gene (*Ek-LotoA*), in our sequenced embryonic transcriptome. This is somewhat surprising given that several Toll (and among them Loto) genes are expressed in arthropods, including *Glomeris*. One reason may be that our data is based on a transcriptome that covers only developmental stages 1 to approximately 22 (as described in Janssen and Budd 2013). It is therefore possible, if not likely, that more Toll genes are expressed at later developmental stages. One of the main functions of Toll genes is their conserved role in innate immune response (Rutschmann et al. 2002; Yokoi et al. 2012), a function that is probably already active during embryogenesis (Jacobs et al. 2014). It is therefore possible that in *Euperipatoides*, an ovoviviparous species, Toll genes are expressed later, close to or after birth, when the juvenile is no longer “protected” by the mother’s immune system. This would also explain why a larger number of Toll genes are active in embryos in the hitherto investigated arthropods (Benton et al. 2016, this study). These embryos all develop outside the mother from eggs that are exposed to pathogens. In that context, it would be interesting to investigate the number and timing of Toll gene expression in the developing embryos of oviparous onychophorans.

### A conserved function in the heart

In *Drosophila*, *Toll* and *Toll*-5 are both expressed in the late developing dorsal tube (the arthropod heart) and thus during the process of dorsal closure (Kambris et al. 2002; Wang et al. 2005). Here, the function of Toll is most likely that of a “critical cell adhesion molecule in the alignment and migration of cardioblasts during dorsal vessel morphogenesis” (Wang et al. 2005). In some of the other investigated arthropods, at least one Loto gene also appears to be expressed in the heart. Examples are *Gryllus Loto7/8/10* and *Parasteatoda LotoA* (Benton et al. 2016, supplementary data). Comparably, late developmental stages for other arthropods including *Tribolium* and the myriapod *Strigamia* are not provided in this paper. Nonetheless, we find expression of *LotoA* in the developing heart of *Glomeris* (Fig. 3), as well as in the onychophorans *Euperipatoides* and *Cephalofovea* (Fig. 2 and Suppl. Fig. S1). Interestingly, in onychophorans, there is a dominant anterior domain of expression in the dorsal region of the head lobes, including part of the frontal appendages (Fig. 2). For another onychophoran, *Peripatoides novaezealandiae*, an enlarged anterior end of the dorsal vessel has been described, “filling the entire space dorsal to the brain” (Pass 1991). In addition, Pass (1991) describes vessels that run from there into the onychophoran frontal appendages. Consequently, it is likely that the described expression of *LotoA* in the dorsal region of the head lobes and the frontal appendages indeed reflects the function of this gene during heart and antennal vessel development.

These data, together with the data from *Drosophila*, and the data provided by Benton et al. (2016), imply a conserved function of Toll/Loto genes in panarthropod heart development, and are fully in line with the description of the circulatory system in Onychophora (Pass 1991).

### Do transverse stripes of expression indicate a conserved role in convergent extension?

A hallmark of Loto class genes that are involved in convergent extension (CE) in arthropods is their expression in periodic transverse stripes (Kambris et al. 2002; Paré et al. 2014; Benton et al. 2016, this study). We find that the single embryonically expressed onychophoran Toll gene, *LotoA*, is expressed in transverse stripes as well (Fig. 2). But does this imply a conserved function of this gene in CE, or any related mechanical process? In comparison to arthropods, in onychophorans, *LotoA* is only transiently and in a dynamic pattern expressed in the ectoderm. The prominent and more persistent transverse stripes of *LotoA* expression are in the underlying mesoderm.

Also, while pair-rule gene orthologs (PRGs) are in control of Loto genes in arthropods (Paré et al. 2014; Benton et al. 2016) (also suggested by conserved gene expression patterns of arthropod PRGs (e.g., Choe et al. 2006; Janssen et al. 2011; Schönauer et al. 2016)), this is unlikely the case in onychophorans, since here, the PRG patterning system is only little (if at all) conserved (Janssen and Budd 2013, reviewed in Janssen 2017). The only PRG that is expressed in the SAZ is *even-skipped* (*eve*), but its expression pattern does not suggest a direct regulation of *LotoA* (cf. the dynamic expression of *LotoA* in the SAZ (Fig. 2 (l–p)) with the static expression of *eve* in the SAZ (Janssen and Budd 2013)).

These differences may be best explained by the different developmental modes of arthropods and onychophorans. In arthropods, all segments (long germ developmental mode) or at least a number of head segments (short germ developmental mode) are patterned directly from the blastoderm, but in onychophorans, all segments form from a posterior-located segment addition zone (SAZ). This onychophoran SAZ differs from that of arthropods. In onychophorans, the SAZ remains more or less the same in shape and size during the process of segment addition, while in arthropods, the SAZ is large in the beginning of segment addition (especially in species that develop a large number of segments during ontogenesis), and subsequently shrinks as its cells are consumed and incorporate into newly forming segments (e.g., Williams and Nagy 2017). Therefore, since there are neither blastoderm-derived segments in onychophorans, nor a shrinking SAZ, there may be no need for CE as it is the case in arthropods.

However, in the onychophoran SAZ and nascent segments, the ectoderm forms as a uniform epithelium, while the mesoderm forms initially as segmental units, the so-called somites (e.g., Mayer et al. 2005 and references therein). These mesodermal blocks change their shape during the process of mesoderm differentiation and organ formation, and it is possible that *LotoA* is involved in this process, a mechanism related to CE. Expression of Loto genes in the early developing mesoderm (or mesoderm progenitors) has been reported for the beetle *Tribolium* (Benton et al. 2016) and the fly *Drosophila* (e.g., for *Toll-2*/*18w*/*tlr*) (Eldon et al. 1994, but see Chiang and Beachy 1994 and Kambris et al. 2002 who do not report on expression of *18w* in the mesoderm). The situation in onychophorans is therefore not necessarily unique in the point that *LotoA* is expressed in the mesoderm. However, the pattern described for onychophorans is clearly different from the expression of Loto genes in the early mesoderm of insects.

Since CE links segmentation (segmental patterning) with AP axis formation in Arthropoda, it is tempting to speculate that a similar interaction is conserved in Onychophora. However, as mentioned previously, onychophoran segmentation is predominantly seen in mesodermal tissue, although the expression of segment polarity genes and Hox genes is conserved in the onychophoran ectoderm (Eriksson et al. 2009, 2010; Janssen and Budd 2013; Franke and Mayer 2014; Janssen et al. 2014). If the interaction of CE-like morphogenetic mechanisms and AP axis segmentation represent conserved features, then the expression of *Euperipatoides LotoA* in the mesoderm may be indicative for mesodermal segmentation in Onychophora. In this context, it has to be said that it has been suggested that mesodermal segmentation may be evolutionary “older” than ectodermal segmentation as seen in arthropods (Budd 2001, reviewed in Janssen 2017), and hence, a Loto-controlled CE-like mechanism may be an ancestral feature of panarthropod body elongation, and that this system has evolved into Loto-controlled CE as seen in Arthropoda (Benton et al. 2016).

## Electronic supplementary material

Fig. S1 (PNG 2.41 MB)

High Resolution Image (TIFF 21863 kb)

Fig. S2 (PNG 1.60 MB)

High Resolution Image (TIFF 25075 kb)

Table S1 (DOCX 13 kb)

Table S2 (DOCX 32 kb)

Table S3 (DOCX 16 kb)

Table S4 (DOCX 42 kb)

Supplementary text 1 (PDF 262 kb)
