# Supplementary figures and images for: Embryonic expression of a Long Toll (Loto) gene in the onychophorans Euperipatoides kanangrensis and Cephalofovea clandestina
Source: Dev Genes Evol. 2018 May 26;228(3):171–8. doi: 10.1007/s00427-018-0609-8 (PMC6013529; doi:10.1007/s00427-018-0609-8)

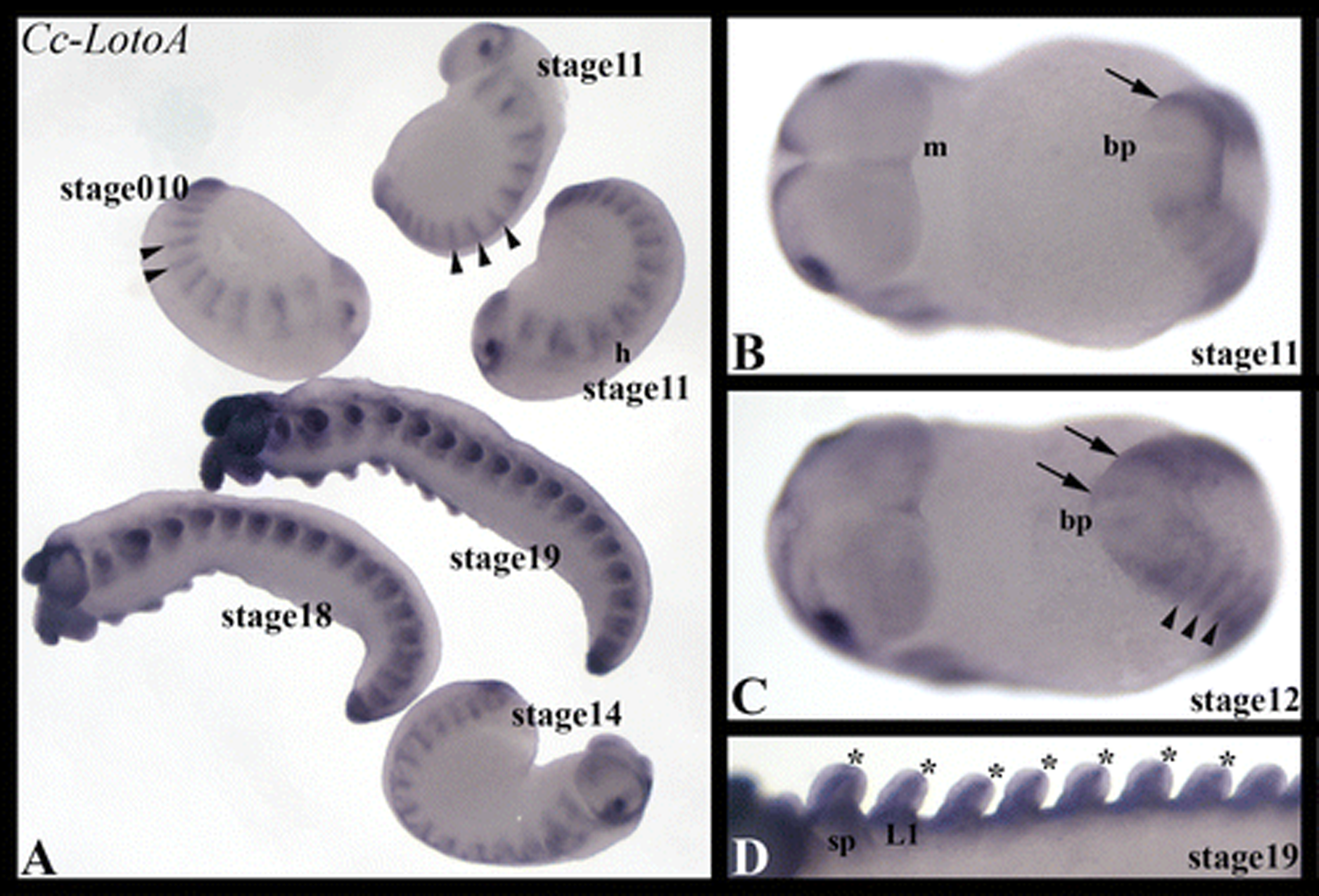

Supplement: Supplementary file 1 — (PNG 2.41 MB) [file 427_2018_609_Fig4_ESM.png]

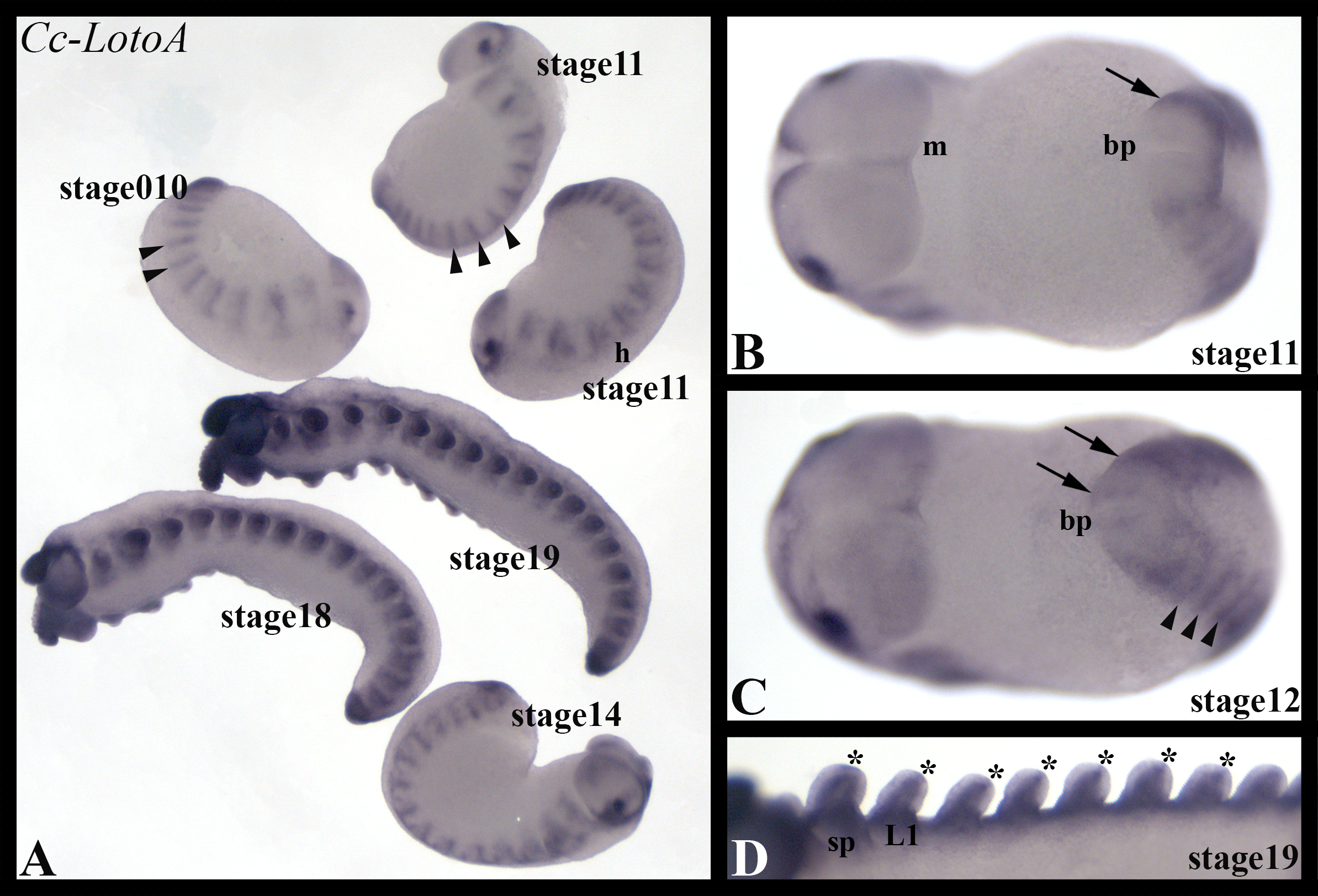

Supplement: Supplementary file 2 — High Resolution Image (TIFF 21863 kb) [file 427_2018_609_MOESM1_ESM.tif]

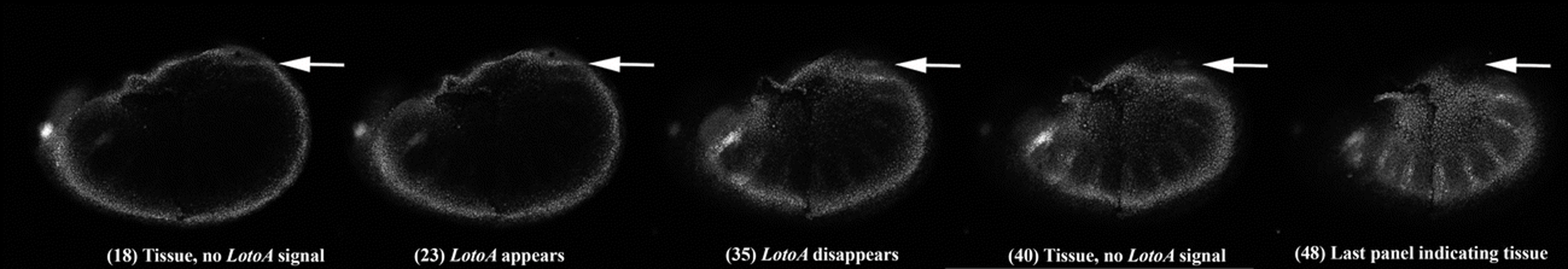

Supplement: Supplementary file 3 — (PNG 1.60 MB) [file 427_2018_609_Fig5_ESM.png]

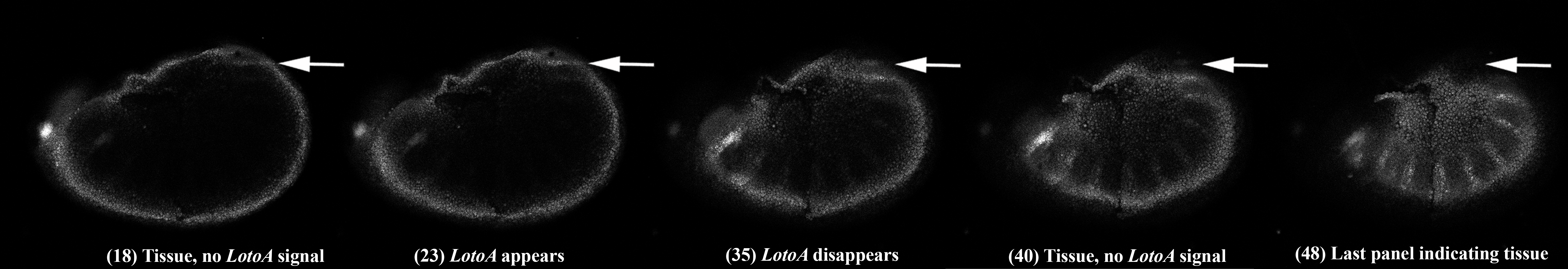

Supplement: Supplementary file 4 — High Resolution Image (TIFF 25075 kb) [file 427_2018_609_MOESM2_ESM.tif]
