# Supplementary material for: Embryonic expression of a Long Toll (Loto) gene in the onychophorans Euperipatoides kanangrensis and Cephalofovea clandestina
Source: Dev Genes Evol. 2018 May 26;228(3):171–8. doi: 10.1007/s00427-018-0609-8 (PMC6013529; doi:10.1007/s00427-018-0609-8)
Supplement: Supplementary file 5 — (DOCX 13 kb) [file 427_2018_609_MOESM3_ESM.docx]

| Primer name | Nucleotide sequence (5’-3’) |
| --- | --- |
| EkLotoA-fw | CTGCCCAAAATTGTAGACCTG |
| EkLotoA-bw | CGTCTTGATCCTTTGGCTTAG |
| EkLotoA-fw2 (used for *Cephalofovea*) | ACTAACATAGTTGATTGCAC |
| EkLotoA-bw2 (used for *Cephalofovea*) | ATCGGGCATAGCGTAACGCA |
| GmLotoA-fw1 | CCAACAACTCGCTGTCCGTCC |
| GmLotoA-fw2 | GACAGACGCTTGGGTGAACG |
| GmLotoA-bw1 | AAGCCCTATCTTCAGCAACCG |
| GmLotoA-bw2 | GCTGCACGGACGAGATGAGG |
| Gm59654-fw1 | TCCTTTGTTGACATGGAGGG |
| Gm59654-fw2 | TTCTGCGATTGCGATACGG |
| Gm59654-bw1 | TCAGTATCTTTCTACCAAATC |
| Gm59654-bw2 | CCGCGGGCATCGCGAAC |
| Gm56792-fw1 | ATGAAGCCAACAACATATTGTAA |
| Gm56792-fw2 | CTCCCCAAATGATTGCAATTC |
| Gm56792-bw1 | TGTTTCCCTCCATGTCAACAA |
| Gm56792-bw2 | GGGAGAGGTAAATATACTTG |
