# Supplementary material for: Embryonic expression of a Long Toll (Loto) gene in the onychophorans Euperipatoides kanangrensis and Cephalofovea clandestina
Source: Dev Genes Evol. 2018 May 26;228(3):171–8. doi: 10.1007/s00427-018-0609-8 (PMC6013529; doi:10.1007/s00427-018-0609-8)
Supplement: Supplementary file 6 — (DOCX 32 kb) [file 427_2018_609_MOESM4_ESM.docx]

| Gene | Accesion number |
| --- | --- |
| *Ek-LotoA* | LT968151 |
| *Cc-LotoA* | LT985877 |
| *Ek-slit* | LT968152 |
| *Gm-LotoA* | LT968147 |
| *Gm-c56792* | LT968148 |
| *Gm-c57369* | LT968149 |
| *Gm-c59654* | LT968150 |
