# Supplementary material for: Embryonic expression of a Long Toll (Loto) gene in the onychophorans Euperipatoides kanangrensis and Cephalofovea clandestina
Source: Dev Genes Evol. 2018 May 26;228(3):171–8. doi: 10.1007/s00427-018-0609-8 (PMC6013529; doi:10.1007/s00427-018-0609-8)
Supplement: Supplementary file 7 — (DOCX 16 kb) [file 427_2018_609_MOESM5_ESM.docx]

| Animal | Gene | Number of LRR (Leucine-Rich Repeat Domain) | Number of TIR (Toll-Interleukin Receptor Domain) |
| --- | --- | --- | --- |
| *Drosophila melanogaster* | Tl6 | 30 | 1 |
| *Drosophila melanogaster* | *Tl2* | 30 | 1 |
| *Drosophila melanogaster* | *Tl7* | 31 | 1 |
| *Drosophila melanogaster* | *Tl8* | 30 | 1 |
| *Tribolium castaneum* | *Tl6* | 30 | 1 |
| *Tribolium castaneum* | *Tl7* | 29 | 1 |
| *Tribolium castaneum* | *Tl8* | 28 | 1 |
| *Tribolium castaneum* | *Tl10* | 28 | 1 |
| *Strigamia maritima* | *LotoA* | 30 | 1 |
| *Euperipatoides kanangrensis* | *LotoA* | 30 | 1 |
| *Euperipatoides kanangrensis* | *Ek-c206929* | 25 | --- |
| *Glomeris marginata* | *LotoA* | 28 | 1 |
| *Glomeris marginata* | *Gm-c57369* | 19 | 1 |
| *Glomeris marginata** | *Gm-c59654* | minimum 7 | 1 |
| *Glomeris marginata** | *Gm-c56792* | minimum 22 | ? |
| *Tribolium castaneum* | *Tc-Tl9* | 19 | 1 |
| *Tribolium castaneum* | *Tc-Tl4* | 22 | 1 |
| *Tribolium castaneum* | *Tc-Tl3* | 21 | 1 |
| *Tribolium castaneum* | *Tc-Tl2* | 21 | 1 |
| *Tribolium castaneum* | *Tc-Tl1* | 25 | 1 |
| *Drosophila melanogaster* | *Dm-Tl9* | 13 | 1 |
| *Drosophila melanogaster* | *Dm-Tl5* | 12 | 1 |
| *Drosophila melanogaster* | *Dm-Tl4* | 12 | 1 |
| *Drosophila melanogaster* | *Dm-Tl3* | 10 | 1 |
| *Drosophila melanogaster* | *Dm-Tl* | 20 | 1 |

LRRseach, default settings, 3 (default FDR); *incomplete sequence information
