# Supplementary material for: Embryonic expression of a Long Toll (Loto) gene in the onychophorans Euperipatoides kanangrensis and Cephalofovea clandestina
Source: Dev Genes Evol. 2018 May 26;228(3):171–8. doi: 10.1007/s00427-018-0609-8 (PMC6013529; doi:10.1007/s00427-018-0609-8)
Supplement: Supplementary file 8 — (DOCX 42 kb) [file 427_2018_609_MOESM6_ESM.docx]

| Stage | Number of investigated embryos | |
| --- | --- | --- |
|  | *Euperipatoides* | *Cephalofovea* |
| 08 | 8 |  |
| 09 | 5 |  |
| 10 | 14 | 1 |
| 11 | 31 | 2 |
| 12 | 24 | 1 |
| 13 | 10 | 1 |
| 14 | 11 | 2 |
| 15 | 6 |  |
| 16 | 2 |  |
| 17 |  |  |
| 18 |  | 1 |
| 19 |  | 1 |

Number of investigated *Euperipatoides kanangrensis* and *Cephalofovea clandestina* embryos stained for *LotoA*
