# Supplementary material for: Embryonic expression of a Long Toll (Loto) gene in the onychophorans Euperipatoides kanangrensis and Cephalofovea clandestina
Source: Dev Genes Evol. 2018 May 26;228(3):171–8. doi: 10.1007/s00427-018-0609-8 (PMC6013529; doi:10.1007/s00427-018-0609-8)
Supplement: Supplementary file 9 — (PDF 262 kb) [file 427_2018_609_MOESM7_ESM.pdf]

Text S2 – Improved in-situ hybridization protocol for

In-situ hybridization protocol for *Euperipatoides kanangrensis* and *Cephalofovea clandestina*

(staining with BMPurple OR FastRed)

H_2O_2 Treatment

Incubate embryos in 2%H_2O_2 in MeOH for 20 minutes at RT; (57µl of 35% H_2O_2 in 1ml MeOH)

Methanol (MeOH) removal

Return embryos to PBS with 0.1% Tween-20 (PBST-0.1%)

- Wash embryos for 5 minutes in 75% MeOH in PBST-0.1%,
- Wash embryos for 5 minutes in 50% MeOH in PBST-0.1%,
- Wash embryos for 5 minutes in 25% MeOH in PBST-0.1%,
- Wash embryos 3 times for 5 minutes in 100% PBST-0.1%

<u>Prehybridization</u>

- Wash in 50% PBST-0.1and 50% HYB-B pH6.5 for 10min
- Replace solution and incubate in 500 µl HYB-B pH6.5 for 5min at 60°C
- Replace HYB-B pH6.5 with (pre-warmed) HYB-A pH6.5 and incubate at 60°C for >2h

<u>Hybridization</u>

- Remove as much HYB-A pH6.5 as possible (without letting the embryos fall dry. Add 50µl of fresh HYB-A pH6.5
- Add 3µl (can also be more or less depending of the concentration/activity) of RNA probe
- Incubate o/n at 60°C

End of Day “ONE”

<u>Probe removal</u>

- Wash 1 x 30 min in Hyb-B pH6.5 at 60°C
- Wash 2 x 15 min 50% formamide in 2XSSC at 60°C
- Wash 1 x 15 min 2XSSCT-0.1% at 60°C
- Wash 3 x 15 min 0,2XSSCT-0.1% at 60°C
- Wash 3 x 5 min PBST-0.1% at room temperature (RT)

<u>Blocking</u>

- Incubate in PBST-0.1% supplemented with 2% sheep serum and 10mg/ml bovine serum albumin (BSA) for 2 hours

Antibody treatment

- Add anti DIG antibody (ROCHE) to a final dilution of 1:2000 in PBST-0.1% supplemented with 2% sheep serum and 10mg/ml BSA
- Incubate for 2-3 hours at RT (on wheel or equivalent)
- Wash several times in PBST-0.1% (wash at least for 1 hour) (on wheel or equivalent)
- Final wash step o/n

End of day “TWO”

- Wash 3 x with PBST-0.1% for 10 min at RT

Detection

- Wash 3 x with staining solution for 5 min at RT

OR

For FastRed staining wash 3 x with 0.1M TRIS pH 8.2 for 5 min at RT

- Incubate in BMPurple (spin for 1min at max speed before use; use supernatant). Centrifuged BMPurple forms less background but stains slower).

OR

For Fast Red, dissolve one tablet in 2ml in 0.1M TRIS pH 8.2 and sterile filter (0.2 µm) before adding the solution to embryos; for elongated staining, exchange FastRed every 6-8 hours.

- Incubate as long as it needs to get a nice staining

Stop staining reaction by washing 3 x 10min with staining solution pH 7.4; add a drop of 37% formaldehyde.

End of day “THREE”

Buffers:

1 X PBST: 80ml 10 X PBS + 8ml 10%Tween20 (for 800ml)

20 X SSC: 70,12g NaCl + 35,28g NaCitrate (for 1000ml)

HYB-A (store at -20C): 25ml formamide + 12,5 ml 20 X SSC + 1ml of 10mg/ml salmon testis DNA + 250 µl of 20mg/ml tRNA + 25µl of 100mg/ml heparin + 5% (=2.5g) dextran sulfate sodium salt + 0,5ml of 10% Tween20; Adjust to pH 6,5; Fill to 50ml with water

HYB-B (store at -20C): 25ml formamide + 12,5 ml 20 X SSC + 0,5ml of 10% Tween20; Adjust to pH 6,5; Fill to 50ml with water

Staining solution: 3ml of 1M Tris pH 9,5 + 1,5ml of 1M $MgCl_2$ + 0,6ml 5M NaCl + 0.06ml of 10% Tween20 (=0.02%); Fill to 30ml with water
